# Monitoring leaf water content with THz and sub-THz waves

**DOI:** 10.1186/s13007-015-0057-7

**Published:** 2015-03-06

**Authors:** Ralf Gente, Martin Koch

**Affiliations:** Faculty of Physics and Material Sciences Center, Philipps-Universität Marburg, Renthof 5, 35032 Marburg Germany

**Keywords:** THz, Sub-THz, Water-status, Water-content, Drought-stress

## Abstract

Terahertz technology is still an evolving research field that attracts scientists with very different backgrounds working on a wide range of subjects. In the past two decades, it has been demonstrated that terahertz technology can provide a non-invasive tool for measuring and monitoring the water content of leaves and plants. In this paper we intend to review the different possibilities to perform in-vivo water status measurements on plants with the help of THz and sub-THz waves. The common basis of the different methods is the strong absorption of THz and sub-THz waves by liquid water. In contrast to simpler, yet destructive, methods THz and sub-THz waves allow for the continuous monitoring of plant water status over several days on the same sample. The technologies, which we take into focus, are THz time domain spectroscopy, THz continuous wave setups, THz quasi time domain spectroscopy and sub-THz continuous wave setups. These methods differ with respect to the generation and detection schemes, the covered frequency range, the processing and evaluation of the experimental data, and the mechanical handling of the measurements. Consequently, we explain which method fits best in which situation. Finally, we discuss recent and future technological developments towards more compact and budget-priced measurement systems for use in the field.

## Introduction

In comparison to other methods like measuring the water potential of the leaves or comparing their fresh and dry weight THz and sub-THz measurements have the advantage of being a non-invasive technique. This means that repeated measurements on the same sample over a long period of time are possible. With invasive techniques such measurements are problematic, because besides the obvious wastage of sample material with each measurement the extraction of tissue from a living organism always causes additional stress. This can obviously affect the result of the experiment.

Electromagnetic radiation in the THz and sub-THz frequency range is strongly absorbed by liquid water [1,2]. Various approaches for biological and medical applications of THz waves exist [3-10]. But the strong attenuation by water often tends to be a problem as samples in this field usually have a rather high water content [11,12]. As a result, this often makes these samples completely opaque for THz and sub-THz waves. Yet, for measuring the water content of a thin sample like a plant’s leaf, the strong absorption turns out to be very convenient [13-21]. Also radiation in the neighboring microwave and infrared range has been used as a tool for water status measurements [22,23]. Often this is done via remote sensing taking several plants at once under observation [24], sometimes even with airborne or spaceborne sensors [25-28]. In contrast to this, the techniques in the sub-THz range (i.e. the upper microwave range) and the THz range, which we will take into focus here, are designed to be used locally on individual plants. Here, we review these new approaches and hope that they will get accepted and widely used by plant physiologists to monitor the water status of plants.

While the dry tissue of the leaf has little influence on the transmitted signal, the attenuation of the signal can be used directly for a qualitative observation of the leaf’s water content. The high contrast between dry biomass and liquid water is caused by the polarity of the water molecules, which results in a high absorption coefficient in the THz frequency range. The capability of this approach for water status measurements was firstly demonstrated by the pioneering work of Hu et al. [13] and Mittleman et al. [14]. In their experiments Hu et al. recorded an image of a freshly cut leaf using THz time domain spectroscopy. In this image the veins of the leaf are clearly visible due to their higher water content and their higher thickness. After two days, the measurements were repeated and an increase of the overall transmission through the leaf was observed showing a decrease of the leaf water content. By performing similar measurements on a leaf of a living plant Mittleman et al. visualized the water uptake of the plant, which was previously subjected to drought stress, after rewatering. In such measurements a sample holder may be needed to keep a leaf in a defined position. Yet, the actual measurement is contact-free, which helps to keep the mechanical stress on the sample to a minimum [3,16,19]. THz and sub THz measurement systems, which are specially adapted for water status detection, are still subject to active research and development [17-19] and not commercially available so far. Yet, several different technical realizations of the underlying idea to use THz or sub-THz waves for water status measurements have been implemented and evaluated. Among these are THz time domain spectroscopy [29-33], THz continuous wave setups [34-38], and sub-THz continuous wave setups [39]. These techniques have different advantages and drawbacks, which make them suitable in different experimental situations. In the following, we will discuss the capabilities of the different approaches and the typical experimental configurations in which they can be used. For each of the different approaches we present experimental data, which demonstrates the capabilities of the technology and might serve as an inspiration for further experiments.

## Review

### Terahertz time domain spectroscopy

**Figure 1:**
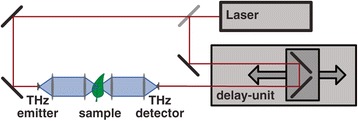
**Schematic of a typical THz time domain setup.** A laser emits short pulses of light, which are used for generation and detection of THz pulses. The delay unit enables scanning across the THz waveform (see also [29]-[33]).

**Figure 2:**
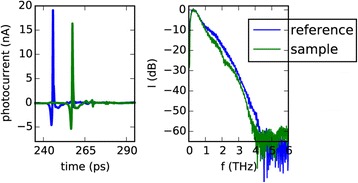
**Example for a THz time domain measurement.** The comparison of reference and sample measurement shows that the signal is attenuated and delayed by the sample. The data was recorded using a laboratory free space setup. To magnify the effect of the sample on the THz pulse, a 7mm thick block of polypropylene was used as a sample in this measurement (see also [29]-[33]).

A typical THz time domain spectrometer consists of several components which serve to generate and detect a short electromagnetic pulse [29-33] and record its time trace. As this pulse typically consists of frequency components from a few hundred GHz to several THz, they are located in the electromagnetic spectrum between microwaves and infrared light. As shown in Figure 1 a central component of such a setup is a laser, which emits short pulses of light with a pulse duration of about 100 fs. A common technique for emitting and detecting THz pulses are photoconductive antennas [40-43]. The light pulses from the laser are used to excite both the emitter and the detector antenna by generating free carriers in the substrate material. At the emitter antenna a bias voltage is applied to accelerate the free carriers. This mechanism generates one terahertz pulse for each incoming light pulse. At the detector, the free carriers, which are generated by the light pulses, allow the terahertz pulses to induce a photocurrent. This photocurrent is measured using a lock-in amplifier or a transimpedance amplifier. In the optical path, which guides the light from the laser to the detector antenna, a delay unit is used to manipulate the time of arrival of the optical light pulses. By using this delay unit it is possible to scan across the THz pulse and record its shape in the time domain. For each measurement with a sample, a reference measurement is performed without the sample to record the characteristics of the measurement setup. Figure 2 shows an example for a THz time domain pulse trace and its representation in the frequency domain. The properties of the sample can be calculated by comparing the results of the sample measurement to the reference measurement. The figure shows how the THz pulse is attenuated and retarded by the sample. Frequency dependent data evaluation is possible by applying a Fourier transform to the time domain data.

A stationary laboratory setup is usually built upon an optical table, which holds the optical components in place. A more flexible and compact alternative are fiber-coupled measurement systems where glass fibers are used to guide the light from the laser to the photoconductive antennas. Moreover, combinations of both techniques are possible. In the following sections we will present different measurement systems, which can be used for different purposes and in different locations.

### Automated long-term experiments

**Figure 3:**
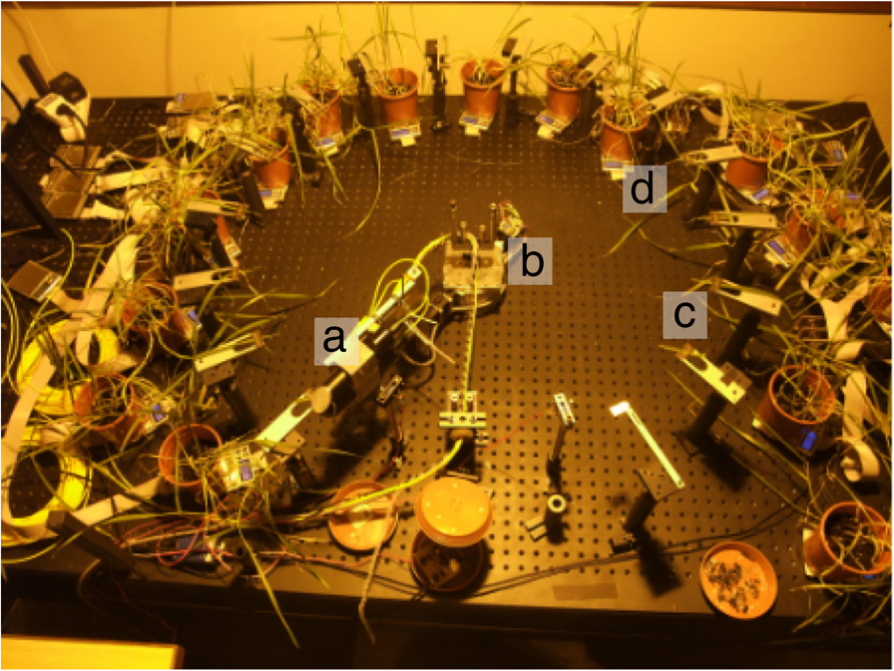
**Photograph of a setup for automated long-term experiments.** The plants (in this potograph: oat, lat. *Avena*) are placed on the table in a circular arrangement similar to the one used by Born et al. [16] to make them accessible to the measurement head **(a)** on the motorized arm of the goniometer **(b)**. One leaf from each plant is placed in a sample holder **(c)**. Below each pot a digital scale **(d)** is placed to keep track of the weight of the pots.

The THz time domain setup shown in Figure 3 is designed for long-term measurement series on a number of plants over a course of several days or even weeks [16]. Once an experiment is started, all steps, which are necessary to carry out the measurements, are controlled by a computer. This way, measurement data can be taken continuously without any need for manual intervention.

The delay unit, which is used here, is built on an optical table as a conventional free space delay line. The remaining optical path to the antennas is fiber-coupled allowing to move the THz antennas and THz optics around on the table. These components are mounted on a measurement head, which can be moved around on a circular route by the motorized arm of a goniometer. Corresponding to that, the plants are arranged on the table in a circular shape and a leaf from each plant is kept in a fixed position by a sample holder. The sample holders are adjusted to be exactly on the circular path of the measurement head. Thus, each leaf can be reached by the measurement head and a fully automated operation is possible. When 15 plants are placed in the setup one roundtrip of the measurement head takes about one hour. By using faster mechanics and data acquisition [44] the speed of the measurements could be increased at least by a factor of 20. Additionally, each pot sits on a computer controlled digital scale to keep track of the pot’s weight as a measure of the amount of water, which is available to the plants. Above the table a high pressure sodium lamp is mounted as a light source for the plants, which is controlled by a time switch.

**Figure 4:**
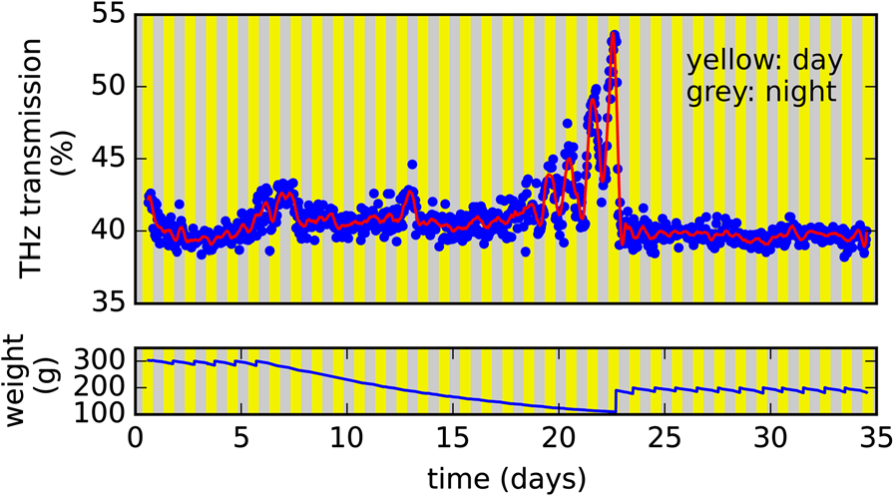
**Result of a measurement series with the automated setup.** The THz transmission through the leaf of a rye plant is plotted together with the weight of the pot. In agreement with Born et al. [16] higher transmission values stand for smaller water content in the leaf. Comparison of the two graphs shows how drought stress builds up while the amount of water available to the plant is decreased. Also the immediate reaction of the plant to rewatering is visible.

Figure 4 shows an example for long-term measurement data, which was recorded using this setup. Over a course of several weeks the water status of rye (*Secale cereale*) plants was observed while they were put under drought stress and finally rewatered. Besides the terahertz transmission the weight of the pots was recorded, too. Measurements were performed approximately once per hour on each of the 15 plants. The plot in Figure 4 shows the results from one of these plants. In the first days of the experiment, the water available to the plants was kept on a constant level. The plants were irrigated daily, which reflects in the sawtooth-like shape of the weight plot in these days. After 6 days, the plants were deprived from water. Comparison of the plot for the terahertz transmission and pot weight shows that it takes several days until the available amount of water is low enough to induce drought stress. The two small peaks in the THz transmission around the 7^th^ and the 13^th^ day of the experiment cannot be attributed to any particular event. The drought stress response starts to become visible on the 19^th^ day. From the 20^th^ day on the drought stress response during daytime is larger than twice the standard deviation of the transmission values before deprivation started (*σ*=0.87*%*). This applies to the difference between day and nighttime, too. We attribute the higher transmission values during daytime to the higher usage of water by the plant during this time. Additionally the opening and closing of the leaves’ stomata might cause a slightly different scattering behavior of the radiation on the leaves’ surface. But this would imply a frequency dependent effect, which has not been observed so far. The usage of water by the plant is constituted by the amount of water which is used for photosynthesis, and the amount which is lost due to physical drying by the incident light. During nighttime the water uptake by the plant from the soil can at least partly compensate for the water loss during the day.

Also the end of the drought stress on the 23^rd^ day is clearly visible. The plot shows that the plant recovers immediately after rewatering and THz transmission comes back to its initial level.

An automated measurement setup like the one described above allows for a variety of experiments, where the development of leaf water content over time is under observation. Possible experiments for the future are the comparison of the behavior of different species of plants under drought stress and the comparison of leaves at different locations within one plant.

### Mobile measurement systems for hand operation

**Figure 5:**
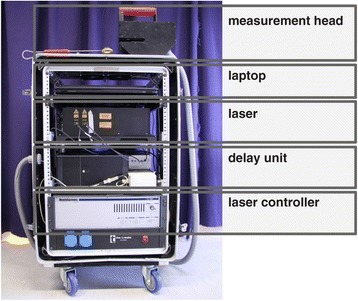
**Mobile THz time domain setup for use in a greenhouse.** All the components of the spectrometer are integrated in a 19" rack case which can be moved around on wheels [45],[46].

Another approach for performing measurements on plants is to bring the measurement system to the plant rather than the plant to the measurement system. To make this possible, the measurement system needs to be a compact, self-contained unit. Figure 5 shows a fiber-coupled THz time domain system, which was designed to be used in a greenhouse. To fit the components of the spectrometer into one 19" rack case a solution for the delay unit had to be found. One possibility is to put a free space delay line in a sealed housing in order to address laser safety regulations and to guard it from dust and other environmental influences. But it is also possible to completely avoid free space optics by using a fiber-stretcher, which periodically stretches and releases several meters of optical fiber and thus generates the optical delay, which is needed for scanning over the THz time domain signal. While a free space delay line can be realized more cost-efficiently a fiber-stretcher allows for higher measurement speed. In either case, the bigger components of the spectrometer, like the laser and the delay unit, are located in the 19" rack case and the THz emission and detection take place in a handheld measurement head, which can be moved to the plant.

This kind of setup allows for a flexible design of biological experiments as plants of different sizes in different locations can be easily reached as long as there is a way to bring the rack case with the spectrometer into a range of about 2 m from the plants. Though, the downside of this concept is that measurements need to be carried out by hand, which can be a time-consuming task depending on the number of plants in the experiment.

**Figure 6:**
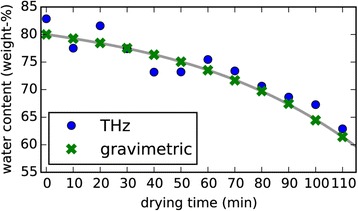
**Comparison of THz measurements and gravimetric measurements of the water content of barley leaves [** 49 **].** For this experiment 10 leaves were detached from a barley plant and their water content was measured repeatedly. Detaching the leaves was only necessary for the gravimetric measurements, which were performed for comparison. The pearson correlation coefficient of the THz measurements and the gravimetric measurements is *r*=0.94. A possible reason for the remaining deviations between the two methods is that a gravimetric measurement gives an average value for the whole leaf while a THz measurement is performed on a small spot. The THz measurements are non-invasive and can also be performed on living plants. The water content was calculated from the THz data using an effective medium model of the leaves [49]. The grey line is a ‘guide to the eye’.

Figure 6 shows a measurement series, which was recorded to compare THz measurements using a mobile THz time domain system and conventional gravimetric measurements. These measurements were performed on ten leaves, which were detached from a barley (*Hordeum vulgare*) plant to make the gravimetric measurements possible. The leaves were dried in an oven. During the drying process they were taken out of the oven every ten minutes and THz measurements and gravimetric measurements were performed. As the THz measurements are non-invasive they are feasible in the same way also on leaves which are still alive and attached to a plant. How the water content is calculated from the THz data is explained in the next section.

### Modeling a leaf as an effective medium

In Figure 4 we have shown that raw transmission values of the THz signal can already act as an indicator for the water status of a plant. But sometimes it’s desirable to know the actual percentage of water in a leaf. Obtaining this value with gravimetric measurements is trivial, but also a destructive method. With sophisticated data analysis it is possible to calculate these values from non-destructive THz time domain measurements. One technique, which aims at this goal, is to use linear transforms like principal component analysis on the measured data [47]. Here, we will have a closer look at another concept, which is based on a physical model describing the transmission of a THz pulse through a leaf [48]. In this model a leaf consists of a mixture of water, dry biomass and air. Using an effective medium theory the dielectric properties of such a mixture can be calculated incorporating the properties of the components and their volumetric fractions. For building such a model the dielectric properties of the components need to be characterized separately. While the values for water and air can be taken from literature [1], for the dry biomass measurements in a laboratory setup are necessary. The absorption coefficient of dry biomass is small compared to the absorption coefficient of water, and thus the effect of the dry biomass on the results of the measurements is small, too. Still, for a reliable model the dry biomass should be characterized separately for each plant species. Jördens et al. [48] use the effective medium theory of Landau, Lifshitz and Looyenga for calculating the permittivity *ε*_*L*_ of the leaf material as follows:

$$ \sqrt[3]{\epsilon_{L}} = a_{W}\sqrt[3]{\epsilon_{W}} + a_{S}\sqrt[3]{\epsilon_{S}} + a_{A}\sqrt[3]{\epsilon_{A}} \;\;, \;\;\; a_{W} + a_{S} + a_{A} = 1 $$

In this central formula of the model, the subindices *W*, *S*, and *A* stand for the three components water, solid matter, and air. One important property of this theory is that it does not make any assumptions about the inner structure of the mixture. When simulating the transmission of THz radiation through a leaf, also its thickness and surface roughness need to be taken into account. For modeling the surface roughness Jördens et al. use a Raleigh roughness factor, which is based on the standard deviation of the height profile of the surface [48]:

$$ \alpha = \alpha_{abs} + \left(\left(\sqrt{\epsilon_{L}}-1\right) \cdot \frac{4\pi\tau\cos(\theta)}{\lambda} \right)^{2} \times \frac{1}{T} $$

In this formulation *α* is a combined expression for the attenuation of the signal by the leaf, which is caused by surface scattering and absorption. *τ* is the the standard deviation of the leaf’s height profile, *θ* the angle of incidence (*θ*=0 for normal incidence), *λ* the free space wavelength, and *T* the thickness of the leaf. Based on this model for the sample material the so called transfer function of the sample is calculated. The transfer function is a frequency dependent representation of how electromagnetic radiation is delayed and attenuated when it is transmitted through the sample. The next step after building such a model is to reverse the problem and extract the model’s parameters from real measured data. This can be done using an optimization algorithm, which fits the model to the measured transfer function [49]. The data in Figure 6, which has been mentioned before, was evaluated using this method.

### Continuous wave THz setups

**Figure 7:**
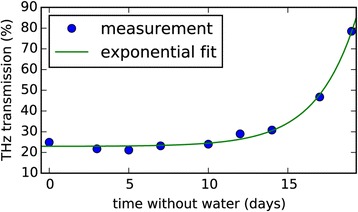
**Water status measurement with a THz continuous wave setup at [** 18 **].** A coffee plant was deprived from water. As drought stress evolves, the Transmission through the leaf is increased (Figure after Kinder et al. [18]).

Instead of short pulses continuous THz radiation can also be used for water status measurements [17,18]. Continuous wave THz setups often use photomixing for generation and detection of the THz waves [38]. The concept is based on overlaying the light from two lasers, which are slightly detuned against each other. When this optical signal is focused onto a photoconductive antenna THz radiation at the difference frequency of the two lasers is generated. Similar to a THz time domain setup a part of the laser light is guided through a delay unit and onto the detector antenna. Similar to the former mentioned methods, the amplitude of the THz signal, which is transmitted through a leaf, is an indicator for the water status of the plant. An example for a measurement series, which was recorded with such a setup is shown in Figure 7. In the experiment shown in the figure, Kinder et al. [18] deprived a coffee plant (*Coffea arabica*) from water and performed measurements with a terahertz continuous wave setup over a course of 20 days. With evolving drought stress the transmission of the THz signal through the leaf increases accordingly.

Lasers for continuous wave generation are usually more compact and cost-efficient than a femtosecond laser. Though, as a usual continuous wave THz setup works at one single frequency, it is not possible to apply the effective medium method here. One possibility to overcome this limitation is to repeat the measurements with different detuning between the two lasers to obtain data for different frequencies. A continuous wave setup can also be operated at several frequencies at once, either by overlaying more than two lasers or by using multimode laser diodes, which emit light at several wavelengths at once [50].

### THz quasi time domain spectroscopy (QTDS)

**Figure 8:**
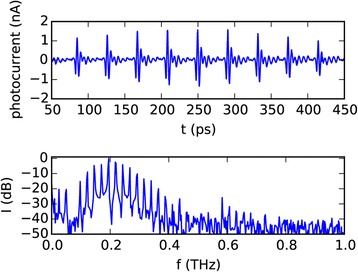
**Signal from a THz quasi time domain setup.** The upper graph shows the signal as it was recorded in the time domain. The Fourier transform of the time domain signal in lower graph reveals that the signal is constructed as the sum of many discrete frequency components [51],[52].

**Figure 9:**
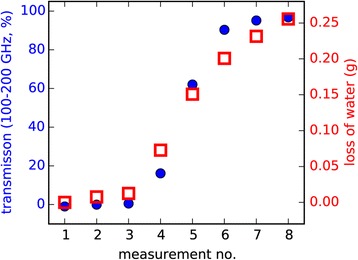
**This experiment shows the feasibility of THz quasi time domain spectroscopy for water status measurements.** A leaf of corn salad was periodically weighed and its THz transmission was measured using a QTDS setup. The results show a good correspondence of water loss and increase of transmission.

A comparably new concept for generating THz radiation is quasi time domain spectroscopy. The setup is almost identical to a THz time domain spectrometer, but the femtosecond laser is replaced by an inexpensive multimode laser diode [51,52]. As such a laser diode generates light at many different wavelengths at once, there is a large number of difference frequencies, which are emitted by the photoconductive emitter antenna. In contrast to a continuous wave setup, which can also be operated with two multimode laser diodes, here the difference frequencies between the different modes of only one multimode laser diode are used. This results in a signal which looks like a train of THz pulses. These quasi pulses are where the name quasi time domain spectroscopy comes from. As shown in Figure 8 the difference frequencies show up in the spectrum of the recorded time domain signal when it is transformed into the frequency domain. THz QTDS setups for water status measurements are still under development, but first measurements, which are shown in Figure 9, show the feasibility of this approach. In these measurements a leaf of corn salad (*Valerianella locusta*) was periodically put in a QTDS setup for measuring the transmission of the THz signal through the leaf. Each QTDS measurement was accompanied by weighing the sample, so the actual loss of water was known, while the leaf was slowly drying. The plot shows a good correspondence of water loss and increase of transmission.

Replacing the expensive femtosecond laser with a cheap multimode laser diode enables QTDS setups to be built much more compact and cost-effectively. Using the QTDS technology, compact, lightweight, battery-powered measurement systems for the use in the field get within reach. New compact, robust, and inexpensive delay concepts like the one proposed by Probst et al. [53] are an important step in this direction.

### sub-THz-measurements

**Figure 10:**
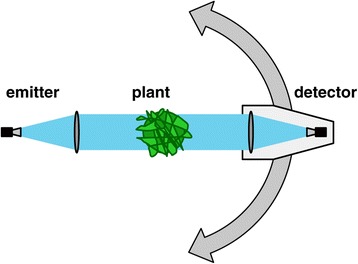
**Setup for sub-THz measurements on plants.** Emitter and the plant are kept in a fixed position. The detector scans around the plant in order to capture radiation, which is scattered by the plant [54].

**Figure 11:**
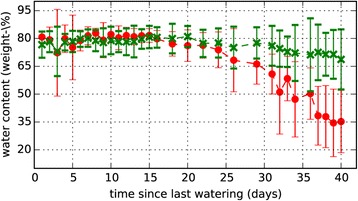
**Result of a sub-THz measurement series on barley plants [** 54 **].** The red curve represents a group of 11 barley plants under drought stress. The green curve shows the corresponding results for the irrigated control group of the same size.

If we move to the upper part of the microwave spectrum, which is also called the sub-THz regime, more powerful emitters are available [39]. The longer wavelengths in the sub-THz regime make it impossible to focus the radiation onto a small spot, but instead the higher power can be used to perform measurements on a bigger part of a plant. For this bigger part an average value is obtained instead of a measurement on one single leaf. When a plant is illuminated by a sub-THz beam with a diameter of several centimeters, a part of the radiation is transmitted straight through the plant, a part of it is absorbed and another part is scattered away in random directions. The scattered part of the radiation is one of the reasons why such measurements are not trivial. The amount and direction of the scattered radiation are determined by the random orientation of the leaves in the illuminated part of the plant. One possibility to take the scattered part of the radiation into account is to capture it by scanning around the plant with the detector [54]. In a setup like the one shown in Figure 10 the emitter and the plant are kept in a fixed position, while the detector is moved on a circular path around the plant. The biggest part of the radiation is transmitted straight through the plant and can be measured when the detector is directly facing the emitter. But still a significant amount of radiation can be detected at other angles besides the direct forward direction. How much of the radiation is scattered also depends on the water content of the plant. Because of this, the relationship between the directly transmitted signal and the scattered signal is nonlinear and the angular scan cannot be replaced by a simple linear proportionality (see Figure 2b in [54]). After integrating over the recorded data, the result of such a measurement is the sum of transmitted and scattered radiation. These values can directly be used as an indicator for the water status of a plant. When additional information about the size and geometry of the plant is available, it is also possible to calculate the actual water content of a plant in percent. Figure 11 shows the results of a measurement series, where one group of 11 barley plants was sufficiently watered throughout the experiment, while another group of 11 plants was deprived from watering. Over a course of several weeks, sub-THz measurements were carried out regularly. The diverging water content of the two groups of plants is clearly visible in the plot. While the water content of the control group stays basically on a constant level, the water content of the stressed group starts to decrease some days after the last irrigation of the plants. The calculation of the water content was done using an effective medium model similar to the one described above. But as measurement values are available for only one frequency, additional information about the size of the plant is required [54]. Sub-THz setups still need to be developed further. Yet, they are good candidates for integration into automated high throughput phenotyping facilities, because no physical contact with the plant is needed for the measurements. The speed of the measurements is limited by the mechanical movement of the detector around the plant and not by the sub-THz technology. Additionally, in such phenotyping facilities additional sensors like cameras or laser scanners, which can be used to determine the size and geometry of the plants, often already exist. While a qualitative assessment of the development of a plant’s water status over time is possible with the raw measurement data the accuracy of the calculated water content strongly depends on the information on the plants size and geometry. Compared to measurements in the THz regime no information on single leaves can be gained. If, for example, a plant gives up only some leaves when drought stress emerges while other leaves are still maintained, this behavior can not be detected using sub-THz measurements. On the other hand, averaging over a bigger part of a plant can also be an advantage, because then the results of the measurements do not depend on the individual variations of only a single or a few leaves, which are picked for the measurements.

## Conclusions

We have outlined the early development and recent advances in the use of THz and sub-THz waves for water status detection. The pioneering work of Hu and Mittleman [13,14] marked the beginning of the development of THz measurement systems for water status measurements. Since then, efforts have been made to make these measurement systems more user-friendly, robust, and cost-efficient, which is necessary for them to be useful in a certain place, e.g. in a laboratory, in a greenhouse or in the field. The current measurement systems already allow for meaningful experiments to investigate the water status dynamics of plants. Further developments will have the aim to bring this technology into the hands of biologists and plant-breeders for their everyday work. In this context, especially the QTDS technology [51,52] is a promising candidate for building compact devices at competitive costs. But depending on the intended use, fiber-coupled TDS systems for automated measurements or sub-THz setups for high throughput facilities can also be a good choice. One important aim for the approaches discussed here is to reveal differences e.g. in the drought stress resistance between different genotypes. In general, this appears feasible. Yet, it is needless to say that the outcome of such experiments will depend on how strongly the drought stress tolerance differs between the genotypes. Variations below 5% in any parameter investigated will be hard to detect. With a broader use of these technologies in plant sciences more experience will be gained concerning measurements on different species as well as the detection of more subtle differences within one species.
